# Regional recurrence of oropharyngeal cancer after definitive radiotherapy: a case control study

**DOI:** 10.1186/s13014-015-0422-8

**Published:** 2015-05-27

**Authors:** Karin Söderström, Per Nilsson, Tina Dalianis, Elisabeth Kjellén, Björn Zackrisson

**Affiliations:** Department of Radiation Sciences, Oncology, Umeå University Hospital, Umeå University, Umeå, Sweden; Department of Oncology and Radiation Physics, Skåne University Hospital, Lund University, Lund, Sweden; Department of Oncology-Pathology, Karolinska Institutet, Karolinska University Hospital, Solna, Sweden

## Abstract

**Background:**

Elective treatment of lymph nodes in oropharyngeal cancer (OPC) has impact on both regional recurrences (RR) and risk of late side effects. This study was performed to quantify the dose-dependent impact on RR and overall survival (OS) in a prospectively collected cohort of OPC from the ARTSCAN study with emphasis on elective treatment.

**Methods:**

ARTSCAN is a previously published prospective, randomized, multicentre study of altered radiotherapy (RT) fractionation in head and neck cancer. In ARTSCAN the elective treatment volume for node positive OPC varied significantly between centres due to local treatment principles. All patients with OPC in complete response after primary treatment were eligible for the present case–control study. Cases were patients with RR during five years follow-up. Patients with no recurrence were eligible as controls. Four controls per case were matched according to T- and N-stage. Mean (*D*_mean_) and median (*D*_50%_) dose for the lymph node level (LNL) of RR in the cases and the corresponding LNL in the controls were analysed with conditional logistic regression. OS was estimated with the Kaplan-Meier method and evaluated by multivariate Cox regression analysis.

**Results:**

There was a dose-dependent risk reduction for *D*_50%_ in the interval that represented elective treatment (40–50 Gy) (OR = 0.18, *p* < 0.05) and a trend in the same dose interval for *D*_mean_ (OR = 0.19, *p* = 0.07). OS rates at five years were 0.39 (0.24-0.65) for cases and 0.70 (0.62–0.81) for controls (*p* < 0.001). The Kaplan-Meier and the Cox regression analysis for cases categorised by delivered dose showed an inverse relationship between dose and survival. The cases with RR in a LNL outside planning target volume (PTV) (*D*_mean_ < 40 Gy) had an OS rate comparable to that of all patients, and those with RR in a LNL in PTV_elective_ (*D*_mean_ 40–60 Gy) or PTV_tumour_ (*D*_mean_ >60 Gy) did significantly worse (*p* < 0.05). The same inverse relationship was also shown for a small subset of patient with known HPV-status, defined by over expression of p16 (*p <* 0.05).

**Conclusions:**

There was a significant risk reduction for RR of elective treatment. However the OS for patients with RR outside target volumes was not affected, with similar results for patients with HPV-positive OPC. This could be an argument for a prospective randomized study on limited elective target volumes in OPC.

## Background

Elective radiotherapy (RT) of clinically negative regional lymph nodes has been part of the standard treatment in head and neck squamous cell carcinoma (HNSCC) for many decades [1, 2]. Several retrospective studies have shown the efficacy of this treatment, but few studies have addressed the dose–response relationships based on the actual dose distribution to the lymph nodes rather than prescribed dose [3–7]. On the other hand, knowledge about relationships between side effects and doses to organs at risk (OARs) is expanding. A number of models that estimate the probability of normal tissue complications link different dose-volume descriptors for OARs with the incidence of specific side effects, for example dysphagia [8–10].

Since the introduction of three dimensional conformal radiotherapy (3D-CRT) and later intensity modulated radiotherapy (IMRT) and volumetric arc therapy with highly conformal dose distributions, it is possible to better spare OARs to reduce side effects [11–14]. One specific sub-group of HNSCC, oropharyngeal cancer (OPC), has undergone a transformation during the last decades. The disease occurs at a younger age and is more often associated with human papilloma virus (HPV) than with tobacco and alcohol abuse [15–19].

As the treatment options evolve and the disease itself changes a reassessment of the current treatment seems motivated.

ARTSCAN was a prospective randomised controlled multi-centre study on accelerated versus conventional fractionation [20]. No significant differences between the fractionation schedules concerning tumour control or late side effects were detected. A large proportion of the patients (48 %) had OPC. At the time of the study there was no clear national consensus on elective treatment. In the study protocol, the elective target volume was specified as “the standard recommendation at each treating institution” with a dose prescription of 46 Gy in 23 fractions, 5 days per week, in both treatment arms. Some centres practiced limited elective treatment with unilateral and selective (not treating all LNL of the neck) treatment and some used bilateral treatment including all LNL of the neck in node positive disease. There was a significant difference, up to a factor of two, between different study sites regarding the volumes prescribed for elective treatment in node positive OPC [21]. Due to this variation in elective treatment volume, not solely dependent on tumour stage, it is reasonable to use this prospectively collected material to estimate a dose–response relationship for regional recurrences corrected for T- and N -stage.

Therefore we studied the dose-dependent risk reduction of neck node recurrences as well as survival based on regional recurrences in patients treated with definitive RT in this prospectively collected cohort of OPC.

## Methods

### Objectives and study design

The objectives of the study were:

1. To compare mean and median dose of a lymph node level (LNL) with a regional recurrence to mean and median dose of the corresponding LNL in matched controls without recurrence. This was carried out as a case–control study.
2. To analyse the impact of regional recurrence on overall survival (OS). This was done both for cases vs. controls and for cases grouped according to mean dose separately.

### Patients

The cohort from which the cases and controls were recruited consisted of all OPC patients with complete response to primary treatment in an earlier reported prospective randomised controlled trial, ARTSCAN [20]. Complete response, in this study, was defined as no residual tumour after primary treatment and no local or regional failure within 6 months after randomisation. The ARTSCAN study was approved by the participating centres’ local Ethics Committees (Regional ethics committee Umeå) and 733 eligible patients with localised HNSCC signed informed consent between 1998 and 2006. About half of them (n = 357) had OPC. Neither chemotherapy nor surgery, other than diagnostic, prior to RT was allowed. Neck dissection as part of primary treatment post RT was allowed according to clinical practice at each participating centre. The use of neck dissection post RT varied between centres where some practiced planned elective neck dissection and some centres used salvage neck dissection in patients with suspected/verified residual neck disease. For additional information on the original study, see [20]. Two of the sites (Stockholm and Umeå) have since the publication of [20] provided information on HPV-status (based on p16-expression as a surrogate marker) for patients with OPC. An extensive quality assurance process for the original study was performed and published [21] with no major trends in the patient characteristics or treatment of OPC during the study period.

Cases for the present study were patients with a regional relapse with or without composite local relapse during follow up. They were identified by the clinical report form and verified in the patient chart. The remaining patients in the cohort with loco-regional control during the follow-up period of five years were eligible as controls. Frequency matching was performed based on T- and N-stage, and to optimise the statistical power of the study, four controls were chosen for each case.

### Radiotherapy

All patients received computed tomography (CT) based 3D-CRT (92 %) or IMRT (8 %) with dose prescriptions according to ICRU [22, 23]. The RT and the quality assurance of the trial have been described in detail elsewhere [20, 21]. Patients included in the ARTSCAN trial were randomised between two fractionation schedules for the planning target volume encompassing macroscopic tumour (PTV_tumour_). The conventional fractionation schedule (CFx) consisted of 2 Gy fractions daily/5 days a week to a total dose of 68 Gy with a total treatment time <7 weeks. Patients receiving accelerated fractionation (AFx) were prescribed 2 Gy fractions daily/5 days a week plus a daily concomitant boost of 1.1 Gy to a total dose of 68 Gy with a total treatment time <5 weeks for PTV_tumour_. As stated in the introduction, all patients had a dose prescription of 46 Gy in 2 Gy fractions daily/5 days a week to the elective PTV (PTV_elective_), the extent of which was not stipulated in the protocol. The major difference between centres in respect to PTV_elective_ was the use of unilateral selective treatment in T1-2 and N1-2b OPC.

### Data collection and localisation of regional relapse

The patients were clinically examined 4–8 weeks after completion of RT to assess treatment result. During the first two years patients were monitored for loco-regional relapse and distant metastases every 3 months, and they were monitored every 6 months thereafter until five years after end of RT. OS was followed through the Swedish population registry.

Treatment plans (CT-images, structures, plans and dose distribution) from ARTSCAN were collected after RT was completed and stored in DICOM-RT format for additional analysis such as the present.

All cases had their charts revised including available radiological imaging to identify the LNL [24, 25] of the regional relapse including side in respect to the primary tumour (ipsilateral vs. contralateral). The corresponding LNL for the controls including laterality was identified. A systematic delineation of the LNL according to [26, 27] was performed by one of the authors (KS) on the original CT-images for all cases and controls. Dose-volume data for these structures were derived from the original DICOM-RT dose files using the software package *RT Bench*™ (Cureos AB, Uppsala, Sweden).

### Dose-volume analysis

We analysed mean dose (*D*_mean_) and median dose (*D*_50%_) for the LNL of relapse in the cases and the corresponding LNL in the controls.

The derived doses for both cases and controls were grouped according to delivered dose to the LNL of interest for the calculation of any dose–response relationships. Four dose intervals were chosen representing LNLs: outside PTV (0–40 Gy), within PTV_elective_ (40–50 Gy), in PTV_elective_ close to PTV_tumour_ (50–60 Gy), and PTV_tumour_ (>60 Gy), respectively.

### Immunohistochemistry of p16

Tumour biopsy sections (4–5 μm) were de-paraffinised and rehydrated, with antigen retrieval in citrate buffer (pH 6) and unspecific binding sites blocked with 1.5 % horse serum in PBS. The sections were then stained with mAb p16^INKA4a^ (clone: JC8, dilution 1:100, Santa Cruz Biotech, Santa Cruz, CA, USA) at +8 °C overnight, before incubation for 45 min. with biotinylated anti-mouse antibody (dilution 1:200, Vector Laboratories, Burlingame, CA, USA). Alternatively, the slides were stained with the CINtec® p16 Histology (805–4713), Ventana Medical Systems Inc., Arizona, USA by following the same protocol with the exception of incubation with the antibody for 1 h at room temperature. For antigen detection, the avidin–biotin–peroxidase complex (ABC) kit (Vectastain, Vector Laboratories, Burlingame, CA, USA) was used. Slides developed in chromogen 3’-diaminobenzydine (DAB) (Vector Laboratories, Burlingame, CA, USA) and counterstained with haematoxylin were then washed and dehydrated, and the cover mounted using VectaMount permanent mounting media (Vector Laboratories, Burlingame, CA, USA). P16 staining was regarded as positive if >70 % of the tumour cells were strongly p16-positive [28, 29].

### Statistical analysis

For univariate analyses the Pearson Chi-Squared-test and Fisher’s exact test were used. The odds ratios for *D*_mean_ and *D*_50%_ grouped according to dose intervals were calculated using conditional logistic regression. Clinically suspected interactions with the dose–response data were also evaluated in the model.

The Kaplan-Meier method was used to estimate OS for all patients calculated from start of RT to death or censoring. Univariate survival time comparisons between cases and controls as well as between cases divided by mean dose to LNL of interest were performed using the log-rank test. Kaplan-Meier survival curves were complemented with a multivariate Cox regression, adjusting for potential confounders. All two-way interaction terms were evaluated in the model.

All tests were two-sided and *p*-value of less than 0.05 was considered statistically significant.

Data were analysed with the statistical software package R (version 2.15.2; R Development Core Team, R foundation for Statistical Computing, Vienna, Austria).

## Results

### Eligibility and patient characteristics

In the cohort of OPC patients with complete response from ARTSCAN, 23 cases with a recurrence in the lymph nodes of the neck were identified corresponding to a regional relapse rate of 7.2 %. Five (22 %) of these regional recurrences had a composite local recurrence. A majority of the cases (95.6 %) and the controls (96.6 %) were in clinical stage III-IV (UICC, Geneva, 1987). 46.6 % of the controls and 43.5 % of the cases were treated with the AFx-schedule. Median age (58 years for cases and 59 years for controls) and other tumour and patient characteristics, including predictive factors reported in [20], showed no significant difference between cases and controls (Table 1). About half of the patients had a neck dissection as part of their primary treatment post RT, and for those the pathology specimen was reviewed for viable tumour (Table 2). In the subset (n = 42) with reported HPV-status (based on p16 expression) there was no significant difference between cases and controls (Table 1). In conclusion, none of the tested two-way interactions differed between cases and controls.

**Table 1:** Patient and tumour characteristics

		Controls	Case	Total	
		n (%)	n (%)	n (%)	*p* value
T	1	19 (21.6)	5 (21.7)	24 (21.6)	1.000
	2	28 (31.8)	7 (30.4)	35 (31.5)	
	3	24 (27.3)	6 (26.1)	30 (27.0)	
	4	17 (19.3)	5 (21.7)	22 (19.8)	
	Total	89 (100)	23 (100)	111 (100)	
N	0	10 (11.4)	3 (13.0)	13 (11.7)	0.069
	1	23 (26.1)	2 (8.7)	25 (22.5)	
	2A	25 (28.4)	4 (17.4)	29 (26.1)	
	2B	15 (17.0)	10 (43.5)	25 (22.5)	
	2C/3	15 (17.0)	4 (17.4)	19 (17.1)	
	Total	89 (100)	23 (100)	111 (100)	
Sex	male	66 (75.0)	19 (82.0)	85 (76.6)	0.584
	female	22 (25.0)	4 (17.4)	26. (23.4)	
	Total	88 (100)	23 (100)	111 (100)	
Hb conc	<140	37 (49.3)	14 (66.7)	51 (53.1)	0.217
	>140	38 (50.7)	7 (33.3)	45 (46.9)	
	Total	75 (100)	21 (100)	96 (100)	
PS *Karnofsky*	<90	6 (7.2)	3 (13)	9 (8.5)	0.465
	>90	77 (92,8)	20 (87)	97 (91.5)	
	Total	83 (100)	23 (100)	106 (100)	
HPV-status (>70 % p16+)	+	28 (82.4)	6 (75.0)	34 (81.0)	0.635
	-	6 (17.6)	2 (25.0)	8 (19.0)	
	Total	34 (100)	8 (100)	42 (100)

**Table 2:** Neck dissection (ND) as part of primary treatment after definitive RT

		Control	Case	Total	*p* value
		n (%)	n (%)	n (%)	
ND	Yes	54 (61.4)	11 (47.8)	65 (58.6)	0.342
	No	34 (38.6)	12 (52.2)	46 (41.4)	
	Total	88 (100)	23 (100)	111 (100)	
If ND, viable tumour present	Yes	15 (27.8)	3 (27.3)	18 (28.1)	0.219
	No	39 (72.2)	7 (63.6)	46 (71.2)	
	Total	54 (100)	10 (100)	64 (100)	

### Dose–response relationships

The dose–response relationships, presented as odds ratios, between cases and controls for the analysed dose intervals including the number of cases and controls analyzed in each interval are presented in Table 3. There was a significant risk reduction for a RR in *D*_50%_ for the interval representing elective treatment (40–50 Gy) and a trend for *D*_mean_ in the same dose interval. We have evaluated the model by adjusting for treatment type and neck dissection without any alterations in the significance levels presented in Table 3.

**Table 3:** Dose–response relationship

Dose interval	OR	*p* value
No of RR/controls		
Mean dose, *D*_mean_		
10–40	0.85	0.856
*5/9*		
40–50	0.19	0.073
*3/27*		
50–60	0.33	0.326
*2/9*		
>60	0.48	0.453
*9/29*		
*Reference = dose category 0–10*		
*4/6*		
Median dose, *D*_50%_		
10–40	0.93	0.935
*4/7*		
40–50	0.18	0.049
*3/34*		
50–60	1.57	0.705
*2/2*		
>60 *9/31*	0.65	0.650
Reference = dose category 0–10		
*4/6*		

Relationship between dose to lymph node level (LNL) of interest and regional recurrences presented as odds ratio (OR). Dose interval presented in Gy, the distribution of cases/controls per dose interval is presented below the dose interval

### Survival

**Fig. 1:**
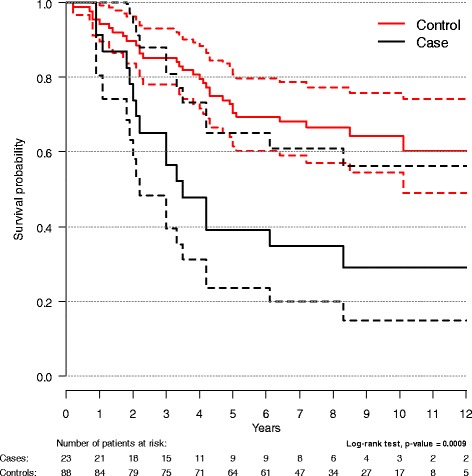
Overall survival in cases and controls. Kaplan-Meier plot of overall survival in cases and controls (95 % CI)

**Fig. 2:**
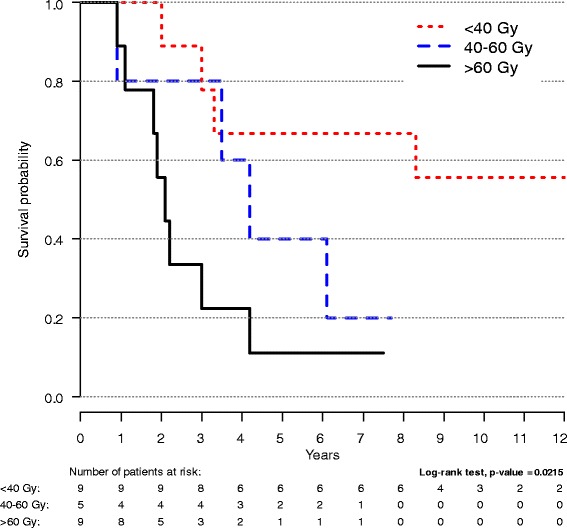
Overall survival in cases based on mean dose (*D*_mean_). Kaplan-Meier plot of overall survival in cases divided according to mean dose to LNL of interest

OS rates (95 % CI) at five and twelve years for the cohort were 0.72 (0.68–0.77) and 0.57 (0.5–0.65). A RR resulted in significantly reduced OS (Fig. 1). The OS rates at five and twelve years were 0.39 (0.24–0.65) and 0.29 (0.15–0.56) for the cases and 0.7 (0.62–0.81) and 0.6 (0.49–0.74) for the controls respectively. The Kaplan-Meier analysis for all cases categorised by mean dose to LNL with RR (Fig. 2) showed an inverse relationship between dose and survival. The OS rates were significantly higher for cases with a relapse in a LNL outside PTV (*D*_mean_ <40 Gy) (0.67 (0.42–1.00)) than for those with a relapse in a LNL in the elective volume (*D*_mean_ 40–60 Gy) (0.40 (0.14–1.00) and in PTV_tumour_ (*D*_mean_ >60 Gy) (0.11 (0.02–0.71). A similar relative inverse relationship was shown for the more limited number of cases with known HPV-status (by p16 over expression in >70 % of the cells) divided in two groups according to *D*_mean_. OS rate at five years was 0.5 (0.25–1.00) for *D*_mean_ <60 Gy and none were alive at 5 years for *D*_mean_ >60 Gy (*p* = 0.046).

Complementing the results presented in Fig. 1, we performed a Cox regression with OS as dependent variable, adjusting for fractionation schedule (AFx vs. CFx) and neck surgery. The variables representing time to relapse and neck surgery were analyzed as a time-dependent (i.e. time at risk is counted from the day of the event until either end of follow up or death). The results are presented in Table 4. No tested two-way interactions were statistically significant. When comparing cases and controls and not taking date of recurrence into account (i.e. not analyzing recurrence as a time-dependent variable) the hazard ratio for cases vs. controls was 2.74 (95 % CI 1.44–5.21). A Cox regression analysis was also performed for cases only, with outcome OS complementing Fig. 2, comparing dose categories (≤40 Gy, 40 ≤ 60 Gy, >60 Gy) adjusted for fractionation schedule and neck surgery (analyzed as a time-dependent variable), Table 5. No tested two-way interactions were statistically significant.

**Table 4:** Overall survival in cases and controls

	HR	CI	*p*-value
Regional relapse	3.56	1.84–6.92	<0.001
Fx-schedule	0.88	0.49–1.59	0.673
Neck dissection	0.85	0.45–1.60	0.619

Cox regression analysis of overall survival in cases vs. controls adjusted for fractionation schedule and neck dissection

**Table 5:** Overall survival in cases based on mean dose (*D*_mean_)

	HR	CI	*p*-value
40–60	1.67	0.35–7.98	0.522
60–80	7.94	1.80–35.04	0.006
Fx-schedule	1.96	0.54–7.17	0.310
Neck dissection	1.83	0.59–5.67	0.298

Cox regression analysis of overall survival in cases categorised according to dose interval in Gy adjusted for fractionation schedule and neck dissection. Dose <40 Gy is the reference category

## Discussion

Our material indicates a risk reduction for regional recurrences (OR < 0.2) in the dose interval corresponding to elective treatment (40–50 Gy) with statistical significance in the logistic regression model for *D*_50%_ and a trend for *D*_mean_. No significant risk reduction for regional relapse was found in our study for *D*_mean_ and *D*_50%_ in the other investigated dose intervals. One explanation for the lack of risk reduction for development of RR in *D*_50%_ and *D*_mean_ >50 Gy could be the close proximity to macroscopic tumour in these LNLs and thereby an inherent higher risk of relapse where a dose <60 Gy might not be sufficient. However, the number of cases and controls in this dose interval is small. A similar dose as suggested in our material has been proposed for elective treatment to the neck in recent studies where an equivalent dose in 2 Gy fractions, assuming α/β = 10 Gy (EQD2_10_) > 50 Gy, does not seem to improve regional control [30, 31], whereas a dose < 30 Gy in 2 Gy fractions has been shown to have a higher regional relapse rate [32]. A prospective randomised trial investigating an EQD2_10_ of 40 Gy to PTV_elective_ presented encouraging toxicity outcome regarding dysphagia, but not yet tumour control data [33].

As the cohort for the present study consisted of patients with complete response after primary treatment, the OS of the cohort was somewhat better than the survival of all OPS presented in [20]. In our study a regional relapse resulted in significantly reduced OS, both at five and twelve years. However, when deciding which LNLs to include in an elective target volume, the consequence on OS of a nodal recurrence outside of the target volume is highly relevant. The effect on OS of regional relapses categorised by delivered dose, however, is sparsely addressed in the literature. This prompted us to do a survival analysis for the cases grouped according to *D*_mean_ representing a relapse outside of the PTV (<40 Gy), in the PTV_elective_ (40–60 Gy), or in the PTV_tumour_ (>60 Gy). A recurrence in PTV_tumour_ has a major impact on OS, whereas a patient with a nodal relapse outside the PTVs has a survival comparable to that of the cohort. Our hypothesis, to this perhaps somewhat contra intuitive finding, is that the relapses in such heavily treated areas are selected to be more treatment resistant than a recurrence in a treatment naive LNL, as well as having more limited treatment options.

The finding that recurrences “out of field” have a survival comparable to the cohort could be an argument for future studies investigating limited elective target volumes omitting treatment to LNL with low risk of relapse in order to avoid late side effects of the treatment.

Since HPV is one of the most important prognostic factors in OPC treated with RT [34–37], we evaluated the OS in the cases with HPV-positive tumours divided according to *D*_mean_ to examine if the inverse relationship between dose and survival seen in all cases holds true for the HPV-positive cases as well. Because of the small number of cases with known HPV-status we restricted the analysis to *D*_mean_ ≤60 Gy or *D*_mean_ >60 Gy in this group. In spite of the small number of cases there was still a significant difference in OS in patients with HPV-positive OPC divided according to dose similar to that shown for all cases. Recently a review of risk factors for loco-regional failure in HPV-associated OPC found that the only factors significant for risk stratification in multivariate analysis apart from smoking were T4- and N3-stages [38], which were evenly distributed in our study between cases and controls. Only five of the HPV-positive cases had available smoking status (all non-smokers). Three of those had *D*_mean_ ≤60 Gy and two had *D*_mean_ >60.

Two different fractionation schedules were used for the PTV_tumour_ in the present study. The ARTSCAN study showed no significant difference in treatment response of lymph node metastases or regional relapses between the fractionation schedules and the use of AFx in our study. Thus the study was well balanced between cases and controls. Therefore no significant difference in outcome depending on fractionation schedule was expected.

Today advanced head and neck cancer is commonly treated with chemo-RT which has shown a survival benefit compared to RT alone [39]. The presented dose–response relationships in this study are investigated for RT without chemotherapy. Since HPV-associated OPC has a favourable outcome, voices are being raised for a possible de-escalation of the treatment to this entity of HNSCC [18, 37, 40]. The incidence of HPV-associated OPC is steadily increasing and now constitutes up to 85 % of all OPC in Sweden [16, 17]. Therefore we suggest that dose–response relationships for RT without chemotherapy are clinically relevant.

According to the ARTSCAN-study protocol, the PTV_elective_ was defined according to clinical practice at each participating centre. At the time of the study there was no clear consensus on what to treat electively in Sweden. This resulted in an up to two- fold variation in the size of the PTV_elective_ with a substantial amount of unilateral and selective elective treatment in the study. Therefore the variation of dose delivered to the LNLs of the neck was only partly dependent on T- and N-stage. This allows analysis of dose–response relationships of regional recurrences corrected for tumour stage. Moreover, the present study was performed on a patient material consisting solely of OPC, which reduces the possible bias of different risk of metastatic spread with respect to primary tumour site.

## Conclusions

Our study shows a significant risk reduction for the development of regional recurrences of elective treatment in the dose levels used in the present study compared to no prescribed elective treatment in OPC. However, in 319 patients treated with RT without chemotherapy, only 23 nodal recurrences occurred during five years of follow-up. Moreover the OS for patients with regional recurrence outside target volumes was not affected, with a similar pattern for a small subset of patients with known HPV-status. This could be an argument for a prospective randomised study on limited elective target volumes to reduce late side effects in OPC.
